# Cytotoxicity of dilutions of bioceramic materials in stem cells of human exfoliated deciduous teeth^*^

**DOI:** 10.1590/1678-7757-2023-0462

**Published:** 2024-08-09

**Authors:** Ana Beatriz Vieira da SILVEIRA, Bárbara Luísa Silva OLIVEIRA, Mariel Tavares de Oliveira Prado BERGAMO, Natalino LOURENÇO NETO, Maria Aparecida Moreira MACHADO, Thais Marchini OLIVEIRA

**Affiliations:** 1 Universidade de São Paulo Faculdade de Odontologia de Bauru Departamento de Odontopediatria, Ortodontia e Saúde Coletiva Bauru Brasil Universidade de São Paulo, Faculdade de Odontologia de Bauru, Departamento de Odontopediatria, Ortodontia e Saúde Coletiva, Bauru, Brasil.; 2 University of Michigan Department of Pediatric Dentistry EUA University of Michigan, Department of Pediatric Dentistry, EUA.

**Keywords:** Biomaterials, Dental pulp capping, Stem cells, Vital pulp therapy

## Abstract

Several materials have been developed to preserve pulp vitality. They should have ideal cytocompatibility characteristics to promote the activity of stem cells of human exfoliated deciduous teeth (SHED) and thus heal pulp tissue.

**Objective:**

To evaluate the cytotoxicity of different dilutions of bioceramic material extracts in SHED.

**Methodology:**

SHED were immersed in αMEM + the material extract according to the following experimental groups: Group 1 (G1) –BBio membrane, Group 2 (G2) - Bio-C Repair, Group 3 (G3) - MTA Repair HP, Group 4 (G4) – TheraCal LC, and Group 5 (G5) - Biodentine. Positive and negative control groups were maintained respectively in αMEM + 10% FBS and Milli-Q Water. The methods to analyze cell viability and proliferation involved MTT and Alamar Blue assays at 24, 48, and 72H after the contact of the SHED with bioceramic extracts at 1:1 and 1:2 dilutions. Data were analyzed by the three-way ANOVA, followed by Tukey’s test (p<0.05).

**Results:**

At 1:1 dilution, SHED in contact with the MTA HP Repair extract showed statistically higher cell viability than the other experimental groups and the negative control (p<0.05), except for TheraCal LC (p> 0.05). At 1:2 dilution, BBio Membrane and Bio-C showed statistically higher values in intra- and intergroup comparisons (p<0.05). BBio Membrane, Bio-C Repair, and Biodentine extracts at 1:1 dilution showed greater cytotoxicity than 1:2 dilution in all periods (p<0.05).

**Conclusion:**

MTA HP Repair showed the lowest cytotoxicity even at a 1:1 dilution. At a 1:2 dilution, the SHED in contact with the BBio membrane extract showed high cell viability. Thus, the BBio membrane would be a new non-cytotoxic biomaterial for SHED. Results offer possibilities of biomaterials that can be indicated for use in clinical regenerative procedures of the dentin-pulp complex.

## Introduction

Human deciduous teeth provide affordable stem cells that can be isolated and cultured *in vitro*. Stem cells from human exfoliated deciduous teeth (SHED) are a highly proliferative population with varied differentiation capacity. As minimally invasive dentistry advocates vital pulp treatments, the understanding of the interactions between pulp cells and capping materials is of paramount importance for tissue engineering, aiding the selection of the best material for clinical treatment.^1-4^

Thus, several materials have been developed to preserve pulp vitality. They should have the ideal cytocompatibility characteristics to promote SHED activity and heal pulp tissue. Calcium silicate-based materials have been used in dentistry due to their ability to stimulate tissue repair by depositing mineralized tissue.^5-7^ Thus, creating a dentin-pulp biostimulation membrane (BBio) is in line with the global trend of development, innovation, and sustainability. The hope is that BBio may be widely indicated for pulp therapy applications.

MTA repair is the most well-established material in the literature. It has properties that repair pulp tissue and induce reparative dentin. However, its setting time and discoloration potential have led the industry to develop new biomaterials. Silicate-based hydraulic cements were then introduced onto the market. Biodentine showed adequate biocompatibility with dental pulp stem cells and TheraCal LC is a resin-modified cement filled with calcium silicate. Furthermore, Bio-C Repair emerges as a new hydraulic cement that is ready to use and, according to the manufacturer, promises excellent consistency and easy application and acts as a barrier against microorganisms, stimulates tissue healing, and avoids contributing to discoloration.^5,8^ The literature still needs more knowledge of these cements as their cytotoxicity to dental pulp cells is yet to be extensively studied. Moreover, the number of studies involving SHED is even more limited.

The International Organization for Standardization^9^has developed guidelines to standardize and reproduce *in vitro* laboratory biomaterial tests. In total, three categories are provided for cellular contact: extracts, direct contact, and indirect contact. Extracts are more advantageous for adherent cells and enable cellular exposure to product concentrations, simulating the diffusion of the substances in tissues, especially when in contact with irrigated tissues.^10^ Measuring cell viability plays an essential role in selecting new materials and providing important initial data prior to conducting clinical trials. A wide variety of assays can detect cell viability based on different cellular functions.^11^

Therefore, cell culture techniques usefully evaluate material biocompatibility and bioactivity. Several studies have quantified the cytotoxicity of capping materials in human dental pulp stem cells from permanent teeth but few have used SHED. Although SHEDs are expected to play a key role in regeneration, knowledge about their responses to materials remains limited.^1,5^

In view of the above, this study aimed to evaluate the cytotoxicity of dilutions of bioceramic material extracts in SHED stem cells and to compare cell viability tests. Its null hypothesis postulated that the materials and dilutions would fail to significantly differ regarding their biocompatibility and cytotoxicity.

## Methodology

### Experimental design

This study included an experimental design involving three study factors (Table 1):

**Table 1:** Factorial study design.

**Treatments**	G1: BBio Membrane (Patent N°.: BR1020170222373)
	G2: Bio-C Repair (Angelus, Londrina, PR, Brazil)
	G3: MTA Repair HP (Angelus, Londrina, PR, Brazil)
	G4: Theracal LC (Bisco Inc., Schaumburg, IL, USA)
	G5: Biodentine (Septodont, Saint-Maur-des-Fosses, France)
**Periods**	24 hours
	48 hours
	72 hours
**Dilutions**	1:1
	1:2

### Ethical considerations

This study was submitted and approved by the Institutional Review Board (protocol CAAE: 59714322.3.0000.5417).

### Sample Selection

SHED from patients who were aged from five to nine years were stored in the institutional biorepository (ethical protocol CAAE 59714322.3.0000.5417) and used in a randomized and blinded way (the researcher that performed the experiment differed from the one who created the biorepository). The experiment was performed thrice in three weeks with samples from a biorepository and the mean value of these measurements was examined according to the obtained results for data normality. The importance of this repetition was to evaluate the reproducibility of the study and to compare the similarity of results. The same experimental design has been previously used.^1,3,12^

Patients’ guardians were duly informed about the risks and benefits of the research and authorized their participation by signing an informed consent form. The children also agreed to participate by an informed consent form.

### Dentin-Pulp Biostimulation Membrane (BBio)

BBio (Patent - No.: BR1020170222373) is a multilayer membrane prepared with equal parts of 1.5 g of chitosan (Sigma Aldrich, St. Loius USA), alginate (Sigma Aldrich, St. Loius USA), and calcium silicate cement (Votorantim-Cimentos, São Paulo, SP, Brazil), according to previous studies.^13-16^ The layers of each material were placed individually and dried inside the oven at 30°C for 15 minutes. Finally, the three-layer hybrid membranes were subjected to final drying for 24 hours and sterilized by ultraviolet light for one hour in a biological cabinet.

### Sample preparation and dilutions

**Figure 1:**
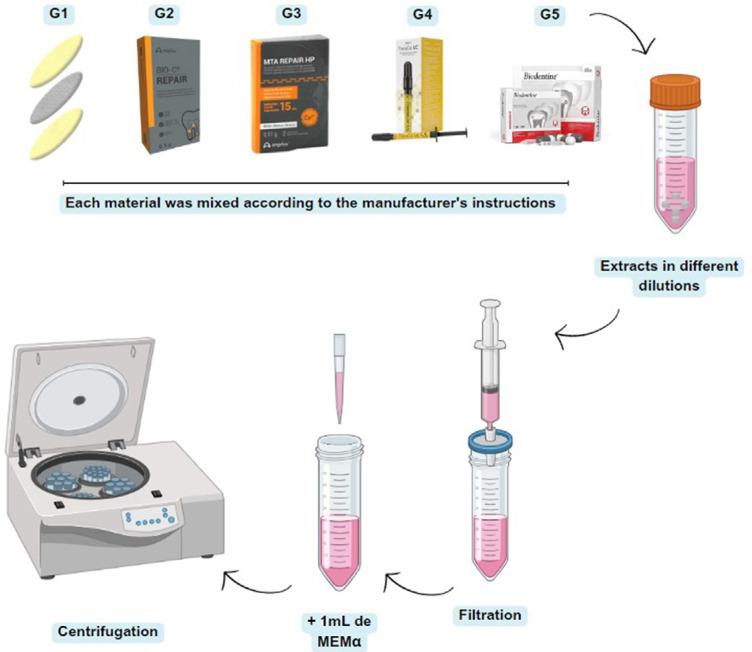
Schema of extract preparation and dilution.

Each material was mixed according to the manufacturer’s instructions. The preparation of the extracts was based on ISO 10993^9^ (2005) and previous studies.^5,6,17,18^ The samples were molded in a sterile cylindrical polyethylene tube (diameter = 5 mm and height = 3 mm). The TheraCal LC was placed in a cylindrical polyethylene tube in 1-mm layer that had been sterilized for 20 seconds (LED-6 Kondortech - 1500MW). Subsequently, the samples were kept in a 5% CO_2_ incubator at 37°C for six hours until they reached the ideal setting. After six hours, the samples were removed from the molds and sterilized by ultraviolet light for one hour in a biological cabinet. Each sample was immersed in 1 mL of medium (αMEM - Invitrogen, Carlsbad, California) + 10% FBS (FBS - Thermo Fisher Scientific, USA) + 1% antimycotic, and an antibiotic solution (Anti-Anti - Gibco, Grand Island, NY, USA) and incubated for three days at 5% CO_2_. After this period, the supernatant was collected and filtered through a sterile 0.22-mm filter (Sigma-Aldrich, St Louis, MO). The supernatant of each material was named as a 1:1 extract + the material name. To obtain the extract diluted at 1:2, another 1 mL of αMEM + 10% FBS was added and named based on the extract 1:2 + and the material name. Before use, all extracts were centrifuged at 4500 rpm for five minutes so all filtered material particles would be deposited at the bottom of the tube (Figure 1).

### Cell viability: MTT assay

Measuring changes in cell viability is critical to assessing cell health. MTT is an accurate, reproducible, and well-defined method in the literature to measure live cell activity by mitochondrial dehydrogenase,^19^ the key component of which is 3-[4,5-dimethylthiazole-2-yl]-2,5-diphenyl tetrazolium bromide. For this trial, SHED was seeded in 96-well plates (1 x 10 ^4^ cells/1 mL of medium per well) and incubated for 24 hours in 5% CO_2_ humidified air at 37ºC for cell adhesion. The positive and negative controls were respectively maintained with αMEM + 10% SFB (αMEM - Invitrogen, Carlsbad, California and FBS - Thermo Fisher Scientific, USA) and Milli-Q Water (Milli-Q^®^ IQ ٧٠٠٣, Merck, Brazil). The extracts were obtained by enriching the cell culture medium with the biomaterial. After filtration and centrifugation, 100 µl were placed in each well with a pipette. At the end of incubation (24, 48, and 72H), the supernatants were discarded, the cells were washed with phosphate-buffered saline, and 110 µl of the MTT solution were added to each well for a final concentration of 0.5 mg/ml. The plates were protected from direct light and incubated at 37º C, 5% CO_2_ for four hours. After this period, the MTT solution was removed and 200 µL of dimethyl sulfoxide (DMSO, Fisher Scientific, Hampton, VA, USA) were added to each well to solubilize the formazan crystals. After 30 minutes, absorbance was measured by an automatic microplate reader (Synergy Mx; BioTek Instruments, USA) and the Gen5 data analysis software at 570-nm wavelength.

### Cell viability: alamar blue assay

Cellular health can be monitored by detecting changes in several key indicators. Resazurin, the active ingredient in the alamarBlue™ Cell Viability Reagent (Thermo Fisher Scientific, Invitrogen, n° DAL1100), is a non-toxic compound that reduces to resorufin when it enters living cells. All the manufacturer’s instructions were followed to conduct the test. The SHED were seeded in 96-well plates (1 x 10 ^4^ cells/1 mL of medium per well) and incubated for 24 hours in 5% CO_2_ humidified air at 37ºC until cell adhesion. At the end of incubation, the cells were exposed to material extracts at 24, 48, and 72H. Positive and negative controls were respectively maintained with αMEM + 10% SFB (αMEM - Invitrogen, Carlsbad, California and FBS - Thermo Fisher Scientific, USA) and Milli-Q Water (Milli-Q^®^ IQ ٧٠٠٣, Merck, Brazil). The extracts were obtained by enriching cell culture medium with the biomaterial. After being filtered and centrifuged, 100 µl were placed in each well with a pipette. After each incubation period with the extracts, the supernatants were discarded, the cells were washed with phosphate-buffered saline, and 100 µl Alamar Blue reagent (1:100) were added to each well. The plates were protected from direct light and incubated at 37º C, 5% CO_2_ for one hour. Density was measured by an automatic microplate reader (Synergy Mx; BioTek Instruments, USA) with Gen5 data analysis software at 560-590-nm wavelengths. The experiments were carried out three times in three independent weeks and the average value of these measurements was examined according to the results obtained in absorbance.^1,12,20^

### Statistical analysis

All experimental conditions were performed in triplicates. The data were subjected to statistical analysis on STATISTICA 10.0 (TIBCO Statistica^®^ ١٤.٠.١.٢٥). The results of cell viability assays were analyzed by three-way ANOVA, followed by the Tukey’s test: Treatments (BBio Membrane, Bio-C Repair, MTA Repair HP, Theracal LC and Biodentine) X Dilutions (1:1 and 1:2) X Periods (24, 48, and 72 hours) (p<0.05).

## Results

### Cell viability: MTT assay

**Figure 2:**
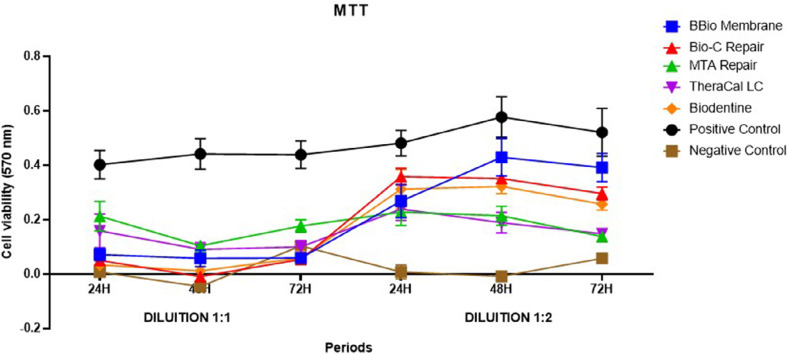
Comparisons between the three factors in cell viability by the MTT test.

An MTT assay analyzed the effects of the bioceramic material extracts on SHED viability and proliferation rates. This study found a statistically significant interaction between its three factors: treatment, dilution, and period (p<0.05) (Table 2 and Figure 2).

**Table 2:** Comparisons between the three factors in cell viability by the MTT test.

	1:1 DILUTION	1:1 DILUTION	1:1 DILUTION	1:2 DILUTION	1:2 DILUTION	1:2 DILUTION
TREATMENTS	24H	48H	72H	24H	48H	72H
	(mean ± SD)	(mean ± SD)	(mean ± SD)	(mean ± SD)	(mean ± SD)	(mean ± SD)
BBio Membrane	0.072 ± 0.023acA*	0.058 ± 0.031abcA*	0.059 ± 0.014aA*	0.268 ± 0.061aB*	0.429 ± 0.069aB#	0.391 ± 0.052aB#
Bio-C Repair	0.050 ± 0.015aA*	-0.010 ± 0.015beA*	0.054 ± 0.014aA*	0.358 ± 0.030bB*	0.351 ± 0.019abB*	0.296 ± 0.024bB*
MTA HP Repair	0.213 ± 0.054bA*	0.104 ± 0.013acA#	0.177 ± 0.023bA*#	0.228 ± 0.049aA*	0.214 ± 0.035cB*#	0.138 ± 0.016ceA#
TheraCal LC	0.159 ± 0.062bcA*	0.091 ± 0.025cA*	0.100 ± 0.023abA*	0.239 ± 0.042aA*	0.189 ± 0.038cB*#	0.148 ± 0.016cA#
Biodentine	0.033 ± 0.010aA*	0.011 ± 0.018bceA*	0.059 ± 0.025aA*	0.312 ± 0.073abB*	0.322 ± 0.027bB*	0.257 ± 0.022bB*
Positive Control	0.402 ± 0.052dA*	0.441 ± 0.056dA*	0.438 ± 0.051cA*	0.481 ± 0.047cA*	0.577 ± 0.075dB#	0.521 ± 0.088dB*#
Negative Control	0.007 ± 0.008aA*	-0.046 ± 0.007eA*	0.104 ± 0.014aA#	0.007 ± 0.026dA*	-0.009 ± 0.006eA*	0.058 ± 0.012eA*

Different superscript lowercase letters in the same column indicate statistically significant differences between treatments at the same period and at the same dilution. Different superscript capital letters in the same line indicate statistically significant differences between dilutions at the same period and in the same material. Different superscript symbols in the same line indicate statistically significant differences between periods at the same period and in the same material. (Three-way ANOVA, followed by the Tukey’s test; p<0.05).

Analysis between groups at 1:1 dilution at 24 and 48 H evinced that the cells in contact with the MTA HP Repair extract showed statistically higher cell viability than the other experimental groups and the negative control, except for TheraCal LC, which showed comparable results to MTA HP Repair (p<0.05). The following experimental groups showed no statistically significant difference with the negative control: BBio membrane, Bio-C Repair, and Biodentine due to their low cell viability (p>0.05). At 48 hours, Bio-C Repair showed high cytotoxicity, with a statistically significant lower means than the MTA HP Repair and TheraCal extracts, in which the latter showed greater cell viability (p<0.05). In all periods, the positive control had statistically greater cell viability than the other groups (p<0.05). At 72 hours, MTA HP Repair again exceeded all materials except for TheraCal. The other experimental groups showed no statistically significant differences (p>0.05). In the analysis over time, at 1:1 dilution, the cells in contact with the MTA HP Repair extract showed greater cell viability at 24H than at 48 and 72H. The other experimental groups showed no statistically significant differences between periods (p>0.05) (Table 2 and Figure 2).

In the analysis between the groups at 1:2 dilution at 24 hours, the SHED in contact with Bio-C Repair extract showed statistically significant greater cell viability than the other experimental groups (p<0.05), except for Biodentine (p>0.05). At 48 hours, the MTA HP Repair and TheraCal LC groups showed statistically significant greater cytotoxicity than the other experimental groups (p<0.05). At 48 hours, BBio Membrane showed promising results, with its highest cell viability results resembling that of Bio-C Repair extract (p>0.05). Finally, at 72 hours, the SHED in contact with BBio Membrane extract showed higher cell viability than the other experimental and negative control groups (p<0.05). Bio-C Repair and Biodentine showed similar values as did MTA HP Repair and TheraCal LC (p>0.05). In all periods, the positive control showed statistically greater cell viability than the other groups (p<0.05). In the analysis over time at the 1:2 dilution, the cells in contact with BBio membrane showed higher viability values at 48 and 72H. MTA HP Repair and TheraCal LC were more cytotoxic at 48 and 72H than at 24H, i.e., cell viability decreased over time. The treatments with Bio-C Repair and Biodentine showed no statistically significant differences between periods (p>0.05) (Table 2 and Figure 2).

The extracts of BBio Membrane, Bio-C Repair, and Biodentine at 1:1 dilution were more cytotoxic than at 1:2 dilution at all periods (p<0.05). On the other hand, at 48H, MTA HP Repair and TheraCal LC 1:2 dilution extracts showed greater statistically significant difference, whereas the results for the other periods showed no statistical differences regardless of dilution (p>0.05) (Table 2 and Figure 2).

### Cell viability: Alamar blue assay

**Figure 3:**
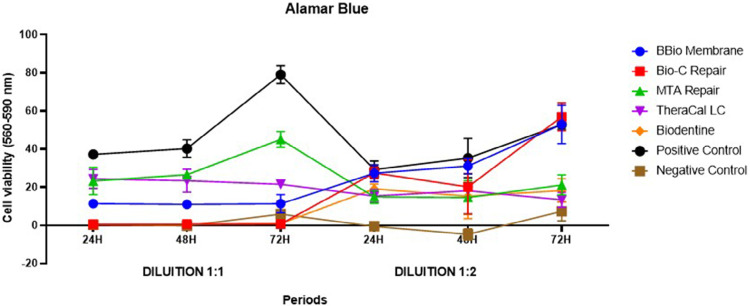
Comparisons between the three factors in cell viability by the Alamar Blue assay.

An Alamar Blue assay analyzed the effects of the different bioceramic material extracts on SHED viability and proliferation rates, finding a statistically significant interaction between its three factors: treatment, dilution, period (p<0.05) (Table 3 and Figure 3).

**Table 3:** Comparisons between the three factors in cell viability by the Alamar Blue assay.

	1:1 DILUTION	1:1 DILUTION	1:1 DILUTION	1:2 DILUTION	1:2 DILUTION	1:2 DILUTION
TREATMENTS	24H	48H	72H	24H	48H	72H
	(mean ± SD)	(mean ± SD)	(mean ± SD)	(mean ± SD)	(mean ± SD)	(mean ± SD)
BBio Membrane	11.346 ± 1.084abA*	11.003 ± 1.900Aa*	11.329 ± 4.824acA*	27.317 ± 4.395abB*	31.112 ± 4.155aB*	52.908 ± 10.078aB#
Bio-C Repair	450 ± 511aA*	588 ± 491aA*	655 ± 532aA*	27.437 ± 3.057aB*	20.181 ± 14.246abB*	56.808 ± 7.314aB#
MTA HP Repair	23.233 ± 7.112bcA*	26.569 ± 1.124bA*	45.005 ± 4.103bA#	14.899 ± 3.409cA*	14.598 ± 8.833bB*	21.166 ± 5.272bB*
TheraCal LC	24.355 ± 5.022cA*	23.456 ± 6.078bA*	21.522 ± 2.281cA*	15.378 ± 3.471bcA*	18.166 ± 3.634bA*	13.402 ± 3.936bcA*
Biodentine	575 ± 568aA*	538 ± 185aA*	870 ± 215aA*	19.174 ± 2.473acB*	15.336 ± 11.863bB*	18.267 ± 6.172bcB*
Positive Control	37.154 ± 2.002dA*	40.210 ± 4.594cA*	78.994 ± 4.687dA#	29.245 ± 4.514aA*	35.260 ± 10.310aA*	52.779 ± 2.572aB#
Negative Control	242 ± 217aA*	-22 ± 752aA*	5.832 ± 6.888aA#	-545 ± 872dA*#	-4.752 ± 2.850cA*	7.296 ± 5.024cA#

Different superscript lowercase letters in the same column indicate statistically significant differences between treatments at the same period and at the same dilution. Different superscript capital letters in the same line indicate statistically significant differences between dilutions at the same period and in the same material. Different superscript symbols in the same line indicate statistically significant differences between periods at the same period and in the same material. (Three-way ANOVA, followed by the Tukey’s test; p<0.05).

In the analysis between groups at 1:1 dilution, SHED showed greater cell viability after contact with MTA HP Repair and TheraCal LC extracts than with other experimental groups (p<0.05) at 24 and 48 hours. At 72H, cells treated with MTA HP Repair showed statistically significant greater cell viability values between experimental groups (p<0.05). In the three periods, the high cytotoxicity of the following materials was relevant: BBio membrane, Bio-C Repair, and Biodentine, which showed no statistically significant difference with the negative control (p>0.05). In all periods, the positive control showed statistically significant greater cell viability than the other groups (p<0.05). In the analysis over time at 1:1 dilution, the cells in contact with MTA HP Repair extract showed greater cell viability at 72H than at 24 and 48H (p<0.05). The other experimental showed no statistically significant differences between the studied periods (p>0.05) (Table 3 and Figure 3).

In the analysis between groups at 1:2 dilution, SHED in contact with BBio Membrane, Bio-C Repair, and Biodentine extracts showed no statistically significant difference with the positive control at 24 hours, evincing positive cell viability (p>0.05). At 48 hours, SHED in contact with BBio Membrane showed better cell viability than the other experimental groups (p<0.05), except for Bio-C Repair, the results of which resembled those of BBio (p>0.05). At 48 hours, the other bioceramic cements showed no statistically significant differences (p>0.05). Finally, at 72 hours, BBio Membrane and Bio-C Repair maintained their pattern and showed no statistically significant differences with the positive control (p>0.05). MTA HP Repair, TheraCal LC, and Biodentine showed no statistically significant differences between them (p>0.05). But at 72H, TheraCal LC and Biodentine showed no statistically significant differences with the negative control, evidencing their cytotoxicity (p>0.05). However, in the analysis over time, the cells in contact with the BBio Membrane and Bio-C Repair at 1:2 dilution showed the highest cell viability at 72h. The other experimental groups showed no statistically significant differences between the studied periods (p>0.05) (Table 3 and Figure 3).

BBio Membrane, Bio-C Repair, and Biodentine extracts at 1:1 dilution showed higher cytotoxicity than at 1:2 dilution in all periods (p<0.05). At 24H, MTA HP Repair showed no statistically significant difference between dilutions. On the other hand, at 48 and 72H, the 1:1 dilution was significantly higher. TheraCal LC showed no statistically significant differences between dilutions (p>0.05) (Table 3 and Figure 3).

## Discussion

The *in vitro* evaluation of the cytocompatibility of a material has served as the first indicator for potential clinical applications. Therefore, selecting a material for vital pulp therapy should consider biocompatibility. These materials should ensure SHED survival and proliferation in the pulp tissue which, in theory, have reparative potential. Current investments in this area include calcium silicate-based cements, which must have bioactive properties by releasing calcium and hydroxide ions to form hydroxyapatite, thus enabling the mineral bond to the inorganic component of dentin.^5,7,8,10^

The literature shows the effects of bioceramic cements with different cell types but few studies have described SHED behavior.^3,6,8,21^ Moreover, this pioneering study with SHED evaluated three study factors: cell viability (BBio Membrane, Bio-C Repair, MTA Repair HP, Theracal LC, and Biodentine), period (24, 48, and 72 hours), and dilutions (1:1 and 1:2). The lack of standardization in extracts preparation motivated this study, which provided a dilution protocol for future research.

Pedano, et al.^7^ (2020) showed that the cell viability and bioactivity of pulp capping materials exposed to human dental pulp cells differ between evaluated materials. This also occurred in this study, the tested materials and dilutions of which showed statistically significant different cell viability, rejecting its null hypothesis.^7^

In this study, at 1:1 dilution, both MTT and Alamar Blue results showed similar behavior for MTA HP Repair and TheraCal LC, i.e., better cytocompatibility than the other experimental groups. These results corroborate a randomized clinical trial, in which TheraCal LC showed a comparable result to MTA HP Repair as a direct pulp capping agent in the primary first molars of 46 healthy subjects aged from five to seven years.^3,22^ On the other hand, the undiluted Biodentine, Bio-C Repair, and BBio Membrane extracts significantly reduced cell viability. Ghilotti, et al.^8^ (2020), evinced a similar behavior between Biodentine and Bio-C Repair, results that corroborate our study. Comparisons with BBio Membrane are more limited because it is a new biomaterial but, according to Benetti, et al.^6^(2019), the undiluted extract of a material can led to cell death, even more so when the properties of this material are poorly known.^6,8^

Previous studies state that, of all sample preparation categories, extracts are advantageous, especially in adherent cell lines as they avoid interfering in cell adhesion and offer good culture conditions. Moreover, the exposure to different concentrations of the material aims to mimic the *in vivo* dilution when in contact with irrigated tissues.^10^ This study found that material cytotoxicity depends on the extract dilution and that most used materials showed decreased cytotoxicity in the more diluted extracts.^6,17,23,24^

In view of these findings, the results of this study at 1:2 dilution differed from those of the total concentrations of materials. At 1:2 dilution, in both assays, Bio-C and BBio Membrane showed greater cell viability than MTA HP Repair, similarly to Ghilotti, et al.^8^ (2020), who used human dental pulp cells and concluded that Bio-C Repair showed excellent cytocompatibility in all tested dilutions (1:1, 1:2, and 1:4), with similar results to untreated cells (control).^8^ Regarding BBio Membrane, previous studies state that chitosan (a component used in the composition of the membrane) in low concentrations offers a safe option with no side effects for human tissues.^13-15^ Thus, the association of chitosan and calcium silicate cement in a pulp capping membrane (BBio) seems to be advantageous for dentistry applications. A previous study with NIH3T3 fibroblasts, which used chitosan membrane associated with other drugs, enabled the viability and proliferation of this cell line, results consistent with those in this study, in which BBio membrane at 1:2 dilution showed better cytocompatibility results with the SHED over time.^13^

Also at the 1:2 dilution, the MTA HP Repair and TheraCal LC cements showed poorer cell viability than the other experimental groups. Interestingly, these materials showed no statistically significant differences in the comparison between the 24- and 72-H period dilutions in the MTT results, whereas MTA HP Repair showed better cell viability results in the 1:1 dilution in the Alamar Blue test. According to previous studies, TheraCal LC is a resin-modified calcium silicate biomaterial that showed negative results in cytocompatibility and bioactivity assays when cultured with SHED.^3,5^. This is explained by its greater inflammatory effect, which, due to its non-polymerized resin monomer, can cause toxicity to the pulp tissue, reducing calcium release. Moreover, as it is the only light-curing material in this study, the generation of heat during light-curing results in unfavorable pulp reactions.^18,25,26^

Biodentine at 1:1 dilution showed exceptionally low or almost absent cell viability. Sequeira, et al.^23^ (2018) used apical papilla cells from third molars and showed that undiluted Biodentine extracts significantly affected cell viability at 24, 48, and 72H. However, when diluted, Biodentine showed satisfactory results at 24H in viability and cell proliferation tests that were comparable with the BBio Membrane and Bio-C Repair but failed to evolve as the other two materials over time. the literature has shown comparable results.^23,27^

The MTT and Alamar Blue assays test intrinsic cytotoxicity by different mechanisms. The MTT (3-(4,5-dimethylthiazol-2-yl)-2,5-diphenyltetrazolium bromide) assay is based on a reducing staining reagent and mitochondrial dehydrogenase activities to determine cell viability by the colorimetric method. On the other hand, in the Alamar Blue (AB) assay, the mechanism of action stems from converting resazurin into resorufin in the cellular reducing environment.^11^ Both tests showed comparable and robust results, Alamar Blue proved to be a more sensitive test, with more evident differences in intra- and intergroup comparisons. However, it offers a good cost-benefit ratio and avoids destroying cells, which can be reused.

*In vitro* assays with cell cultures have helped the understanding of the mechanisms involved in biological responses. The literature includes a few similar studies but most use other types of cells and analyze only a new bioceramic cement, ruling out comparisons between them and dilutions.^6,17,24^ Therefore, the main difference of this research refers to its use of stem cells from primary teeth, offering new alternatives for bioceramic materials in pediatric clinics. However, this study has some limitations. Although its results evade direct application to clinical cases in humans, they are scientifically significant because they represent an appropriate prototype for evaluating several initial attributes of dental materials. Thus, further research using *in vivo* animal models is needed to confirm the results of this trial.^7,10,28,29^

## Conclusion

This study reported the cytotoxicity of different dilutions of bioceramic material extracts in SHED. MTA HP Repair showed the lowest cytotoxicity even at a 1:1 dilution. At a 1:2 dilution, SHED in contact with the BBio membrane extract showed high cell viability. Thus, the BBio membrane would configure a new non-cytotoxic biomaterial for SHED. The results of this study offer biomaterial possibilities that can be indicated for use in clinical regenerative procedures of the dentin-pulp complex. Furthermore, both viability tests proved to be reliable and robust in the analyses.
